# Work-related musculoskeletal complaints among haemodialysis nurses: An exploratory study of the work situation from an ergonomic perspective

**DOI:** 10.3233/WOR-205241

**Published:** 2022-07-15

**Authors:** Eva Westergren, Magnus Lindberg

**Affiliations:** Department of Caring Sciences, Faculty of Health and Occupational Studies, University of Gävle, Gävle, Sweden

**Keywords:** Haemodialysis, nurse, work-related musculoskeletal disorders, ergonomics, reflections, qualitative research

## Abstract

**BACKGROUND:**

Haemodialysis nurses have a high prevalence of musculoskeletal complaints, but the reason for this is yet unknown.

**OBJECTIVE:**

The aim of this study was to carry out an exploratory analysis of the work situation of haemodialysis nurses from an ergonomic perspective.

**METHODS:**

Non-participant observations and reflective discussions to assess the work environment and explore possible potential hazards contributing to musculoskeletal complaints were conducted among nineteen nurses at five haemodialysis centres. Additional reflective notes from the observer’s experiences and progress in the field were made. Analytic integration was applied to merge the collected data.

**RESULTS:**

Eight haemodialysis work tasks believed to increase the risk of developing work-related musculoskeletal complaints were identified. Different types of musculoskeletal complaints, mainly in the upper extremity, were mentioned. The design of the dialysis machine used and the physical demands of repetitive work procedures were implicated.

**CONCLUSIONS:**

Our findings add to the understanding of the work-related factors that contribute to musculoskeletal complaints among haemodialysis nurses. The findings support the hypothesis that there might be an association between materials used and the development of work-related complaints involving the fingers, hands and wrists of this population. Repetitive work tasks that have an impact on the development of musculoskeletal problems need to be further investigated.

## 1 Introduction

Musculoskeletal complaints are common among persons working in the nursing profession [1, 2], and as many as nine out of ten haemodialysis nurses experience musculoskeletal problems in some part of their body. The prevalence of musculoskeletal hand pain among haemodialysis nurses stands out in comparison to the prevalence of similar hand pain among other nurses working in a hospital setting [3] and to workers in general who perform repetitive tasks with their hands and arms [4]. Prolongated musculoskeletal pain that progresses into chronic pain can often lead to impaired work ability and disability. Despite musculoskeletal pain, many people continue to work although their self-rated work ability was decreasing [5, 6]. About one in ten haemodialysis nurses have been absent from work within the previous 12 months due to hand pain, but many continue to work. Every second haemodialysis nurse on duty reports having hand complaints [3].

There are ergonomic risks associated with in-patient nursing care tasks [7]. Nursing tasks performed more than 10 times a day have been found to increase the probability of developing work-related musculoskeletal problems [8] e.g. wrist and hand pain [9]. Repetitive and forceful hand activities are among the work-related factors haemodialysis nurses are being exposed to in their daily work. The reason for the high prevalence of musculoskeletal complaints among haemodialysis nurses is yet unknown. It has been hypothesized that there might be an association between the type of machines and disposable materials used and the occurrence of hand complaints, but this was not empirically supported [10]. As we have failed to identify any studies exploring the work environment’s role in the development of musculoskeletal complaints among haemodialysis nurses, this study was designed to identify the work activities the nurses believe could increase their risk of developing musculoskeletal complaints. The aim of this study was to carry out an exploratory analysis of the work situation of haemodialysis nurses from an ergonomic perspective.

## 2 Methodology

A descriptive, exploratory qualitative approach was applied to research the haemodialysis nurses' physical work environment that has not been thoroughly investigated in the past. Data were collected from 2 May 2019 to 14 June 2019.

### 2.1 Research ethics

The study protocol was approved by the Regional Ethical Review Board in Uppsala (registration number 2017/229). All of the participants received written information about the aim of the study and its procedures in advance. The information was repeated verbally at the time of data collection. All nurses participated voluntarily.

### 2.2 Study population, sample size and context

To appraise the work environment and explore any potential hazards that could lead to the development of musculoskeletal complaints among haemodialysis nurses, 19 nurses (17 females) were observed at five haemodialysis centres in Sweden. Additionally, 2–6 nurses from each centre, together with the observer, participated in a reflective discussion concerning ergonomics in the workplace and work-related musculoskeletal complaints. The three most common haemodialysis machines in the country were used for the haemodialysis treatments. Haemodialysis treatments were performed on 22–54 patients 3–5 times per week, and each treatment lasted about four hours. During a shift, the 6–10 nurses working in the centre were divided into 2–4 teams that cared for 14–26 haemodialysis patients. The patients were located in 3–8 treatment rooms. Since there were nurses training to be haemodialysis nurses at the time of the data collection, there were more staff than usual. The observed nurses had a mean age of 45.3 years (SD 10.9, range 28–65). They had worked as a nurse for an average of 16.3 years (SD 9.3, range 3.5–36) and as a haemodialysis nurse for an average of 9.2 years (SD 8.0, range 0.5–25). The rate of full-time employment at the time averaged 90.5 % (SD 10.3, range 80–100). Of the 19 observed nurses, 10 reported that they experienced daily work-related musculoskeletal pain from their hands, fingers, and wrists.

### 2.3 Data collection

Non-participant observations, reflective discussions and reflective notes documenting the haemodialysis nurses' work components were used throughout the haemodialysis process to collect the data. In the non-participant observations, a single observer (first author) gathered data regarding the physical setting, the participant’s activities and interactions, frequency and duration of performed activities, and postures used in completing the various tasks. Accordingly, the observer did not participate in the care activities to prevent alterations in the working conditions, but was present to take notes about what was happening and how the nurses acted during the work activities. The observations were, however, somewhat more attentive to manual activities that are known to expose workers to an increased risk of musculoskeletal injuries in the distal upper extremity [11]. The observations were conducted during the daytime shift between 07:00 and 15:00 for three consecutive workdays at each centre, which resulted in a total of 112 h.

Reflective discussions were initiated based on what was observed during the observations to gain an understanding of what the nurses experienced as something that could have an impact on musculoskeletal complaints. The reflective discussions were conducted with the observed nurse and also with any colleagues involved so they could give input from their perspective in regard to interaction, support, working positions and body language/movements during the work situation and/or work tasks. The discussion was based on how the situation affected the nurse’s posture, body language and the workspace. To gain clarity regarding the work procedure and potential hazards, the observer asked reflective questions that added to the discussion. Examples of reflective questions are: Please describe your experience of that component of the procedure in regard to workload. Please describe how you perform that component of the procedure. I can see that you need to use some effort to perform that component of the procedure, how would you rate the intensity of the effort you have to apply using this scale (Borg CR-10 scale)? An example of a more specific reflective question asked is: Which part of your hand/fingers do you think you are straining when you connect and disconnect the bloodlines?

Reflective notes documenting the researcher’s personal reflections, experiences and progress in the field were made by the observer who is a registered nurse with substantial experience in haematology and nephrology nursing, but not in the haemodialysis setting. The observer has good knowledge of the musculoskeletal system, its functions and diseases. She is also an expert in how to make adaptations in the workplace that can lower the risk for health problems and accidents as she has regularly taught these subjects the past 15 years at a university. However, this was the first time she systematically collected ergonomic related data. The field notes were made while still on location at the haemodialysis centres and concerned observational strategies, ergonomic challenges during haemodialysis procedures as well as personal experiences and emotions. The reflections focused on the ergonomic challenges and the differences between the described perceived tasks and the observed execution of the tasks with the haemodialysis equipment.

The field notes and reflections consisted of written descriptive notes. At the end of the observation day, the notes were read and reread, and additions were made as needed. They were then read again to ensure the content reflected the observed components of the haemodialysis procedures as well as how each task affected the nurse from an ergonomic perspective.

### 2.4 Data analysis

The analytic goal was to integrate the collected data by intentionally merging the findings from the observations, reflective discussions and notes in the results to expand the understanding of the haemodialysis nurses’ work situation from an ergonomic perspective. At first, the data from each centre were analysed separately in order to find topics and codes meaningful to the aim of the study. In this process, the transcribed data were read and compared several times. Thereafter, the sets of topics and codes from the centres were compared and contrasted in a matrix to explore in which way the findings from the observations, discussions and notes confirm, disconfirm, qualify, or expand each other. A simplified illustration of the analysis matrix is presented in Table 1. Narrative presentations were finally generated from the matrix comparisons to display the integrated results.

**Table 1:** Example of the summary matrix used in the analysis procedure

Work task related to increased risk of musculoskeletal complaints	Topics or codes from data collection		
	Observation	Reflective discussion	Reflective notes
Flushing accesses	Before and after every single treatment, the functionality of the access was checked by flushing 0.9% normal saline. A filled luer-lock 10 mL syringe was attached to each lumen. Number of flushes varied substantially (1–15 flushes/lumen) between the observed cases. The nurse has a standing or sitting position and various postures and twisting is observed.	The work task was done to secure access patency. Standing or sitting position was dependent on type of access and personal preference. Nurses experience high physical demand on the thumb, index and middle fingers.	The patient’s safety is put before the ’ nurse’s own physical working position. The nurses height and dexterity could be related to unfavourable postures. The number of flushes poses a risk.

## 3 Results

**Fig. 1:**
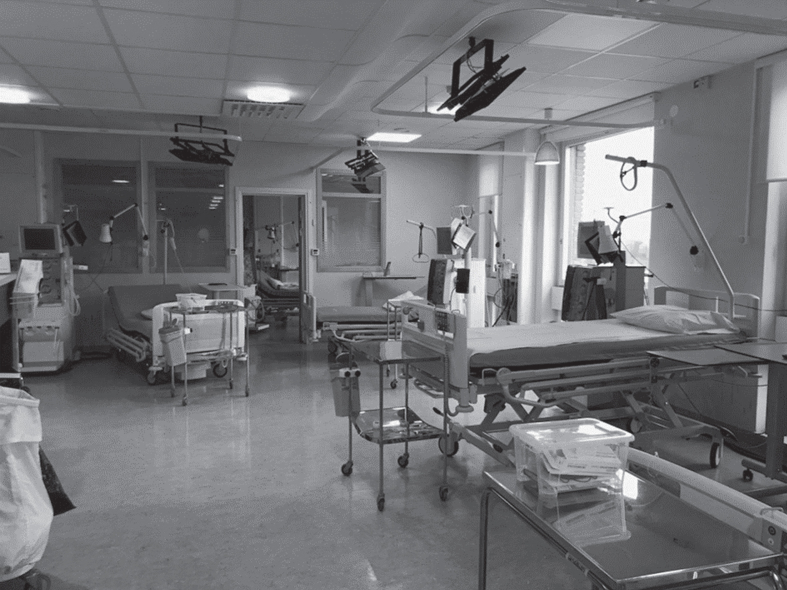
Physical layout of a haemodialysis room from a Swedish centre. Used with permission from the hospital administration.

In Table 2 is a general description of the workflow during a haemodialysis nurse’s daytime work shift, along with the activities observed. The physical layout of Swedish haemodialysis centres vary, but one typical treatment room is shown in Fig. 1.

**Table 2:** Example of the workflow experienced by a haemodialysis nurse on a typical workday

Time frame	Activity	Main body	Activities
		position	observed
07:00–07:15	Attends staff meeting that allocates tasks for the day and checks the medical records for the day’s patients	Sitting	No
07:15–07:30	Sets up and primes a haemodialysis machine	Standing	Yes
07:30–07:45	Connects the first patient to the haemodialysis machine, which can include drawing blood sample(s) or performing other diagnostic tests prescribed by the physician	Sitting or standing	Yes
07:45–09:00	Sets up and primes additional machines required for additional patients	Standing	Yes
	Sequentially connects arriving patients (every 15–20 minutes) to the haemodialysis machines, which can include drawing blood sample(s) or performing other diagnostic tests prescribed by the physician	Standing or sitting	Yes
	Performs routine checks of patients undergoing haemodialysis every 30 minutes	Standing	Yes
09:00–09.20	Break for coffee/tea	Sitting	No
09.20–11.00	Performs direct patient care such as: patient education, preventative or actual treatments related to complications, routine checks of patients undergoing haemodialysis every 30 minutes, and serving beverages, sandwiches and nutritional drinks to patients	Standing	Yes
	Indirect patient care; documentation, care plans and other records	Sitting	No
	Refills supplies at work station	Standing	Yes
11.00–11.45	Lunchbreak	Sitting	No
11.45–13.45	Sequentially disconnects patients from the haemodialysis machines (every 15–30 min)	Standing or sitting	Yes
	Removes disposables and cleans all reusable equipment	Standing	Yes
	Prepares for the next shift by setting up haemodialysis machines	Standing	Yes
13.45–14.00	Break for coffee/tea	Sitting	No
14.00–15.00	Completes medical record entries of finalised haemodialysis sessions	Sitting	No
	Assists colleagues from the afternoon shift connect the patients that arrive for their haemodialysis treatments, which can include drawing blood sample(s) or performing other diagnostic tests prescribed by the physician	Standing or sitting	Yes
15:00	End of observations

### 3.1 Activities with increased risk of work-related musculoskeletal complaints

Eight work tasks (Table 3) believed to increase the risk of developing work-related musculoskeletal complaints were identified. When starting the priming procedure, the nurses described different types of musculoskeletal complaints/symptoms in their fingers, hands, wrists and shoulders depending on the design of the dialysis machine used. From the observations, the priming procedures were accomplished in a standing position with short forward bending sequences that lasted 1–2 s. The degree of bending depended on the nurse’s height. The observations also revealed that when connecting and disconnecting the bloodlines and the dialysate inflow and outflow hoses to the dialysis filter, there was a resistance that required greater hand strength. When the disposable bloodlines were screwed onto the filter, cracking sounds were audible. Depending on the position and angle of the hand while assembling the lines, physical demands were placed on different parts of the hand, i.e. the thumb and scaphoid joint, index finger, middle finger, wrist or metacarpals. Several nurses told that they experienced fatigue in their fingers and in the musculature and tendons on the back of their hand after priming several machines. They said that it was related to their having to grip and pull back with two fingers (index and middle finger) against the palm of their hand on the dialysate connectors to disengage them. Here the nurses also described how the calcium content of the water contributed to the difficulty. With limescale, the resistance was increased when attaching and detaching the connectors. In addition, the nurses told how the removal or tightening of protective caps and the opening of packages containing disposable materials was physically demanding for their fingers.

**Table 3:** Eight work tasks believed to be associated with the development of work-related musculoskeletal problems

Workflow segment	Work task	Related musculoskeletal complaints	Self-perceived degree of intensity of physical effort (Borg CR-10 scale) Median/IQR
Setting up/priming	Connecting the difficult to connect bloodlines and dialysate hoses to the filter (*n* = 105)	Fingers, hands, wrists	3.00/2
The haemodialysis treatment	Flushing accesses (*n* = 212)	Fingers	3.00/0
The haemodialysis treatment	Opening and closing stiff clamps on bloodlines (*n* = 199)	Fingers	3.00/1
The haemodialysis treatment	Retightening of bloodline connections (*n* = 185)	Fingers, wrists	3.00/1
The haemodialysis treatment	Working with raised arms (*n* = 16)	Arms and trapezius muscles	3.00/1
The haemodialysis treatment	Compressing cannulation sites (*n* = 43)	Fingers, arms, shoulders, and back	3.00/5
After concluding the treatment and preparing for the next one	Cleaning (*n* = 54)	Hands and wrists	3.00/2
The physical environment	Moving equipment and machines (*n* = 6)	Torso and back	3.00/2

IQR = Inter quartile range.

**Fig. 2:**
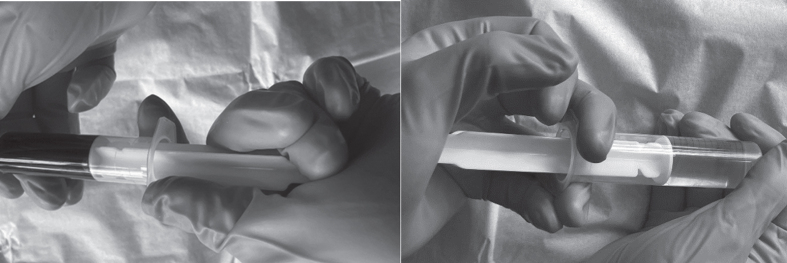
Reconstruction of the flushing process performed on the central venous catheter before connection to the bloodlines. Blood and heparin are aspirated from the central venous catheter before flushing the lumen multiple times with 0.9% normal saline.

A well-functioning vascular access is necessary to connect the patient to the haemodialysis machine. The accesses used were either a central venous catheter (CVC), arteriovenous fistula (AVF) or arteriovenous graft (AVG). The accesses were checked before the start of the dialysis treatment with a flushing process. At the different dialysis centres there was a variation in the number of times the access was flushed with normal saline. An observed 1–15 flushes per lumen were performed before the bloodlines from the machine were connected. Flushing generated a somewhat hard physical demand on the thumb, index finger and middle finger of the hand being used. This flushing process is shown in Fig. 2.

**Fig. 3:**
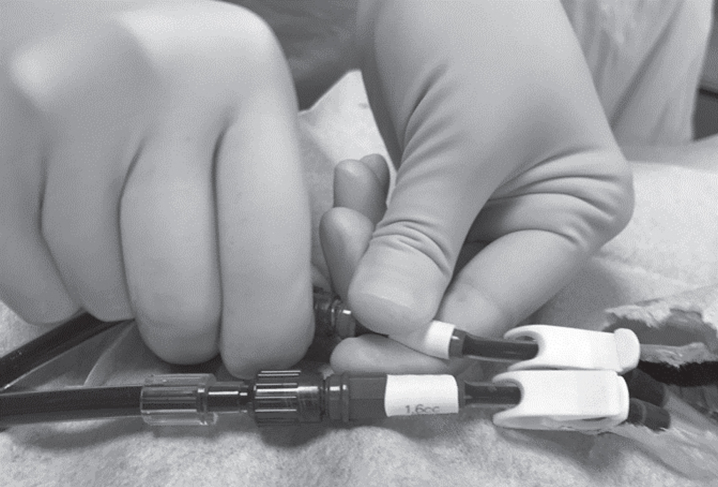
Connecting the bloodlines to a Central Venous Catheter.

Connection of the bloodlines to an AVF or AVG was accomplished by a nurse working alone or together with a colleague. When connecting the bloodlines to a CVC (Fig. 3), the nurse was in a standing position, often leaning slightly forward about 130° towards the patient. This was also performed alone or together with a colleague. When this was carried out by two persons, the assistant would help the nurse with e.g. filling the normal saline syringes and handling the gauze pads with disinfectant in order to maintain the most aseptic technique possible. When the nurse performed this procedure alone, more planning and preparation was required so that the necessary items were within reach. Work done alone generated working positions with more twisting of the back and shoulders as well as stretching of the arms in order to reach the materials being used, than work done in pairs.

**Fig. 4:**
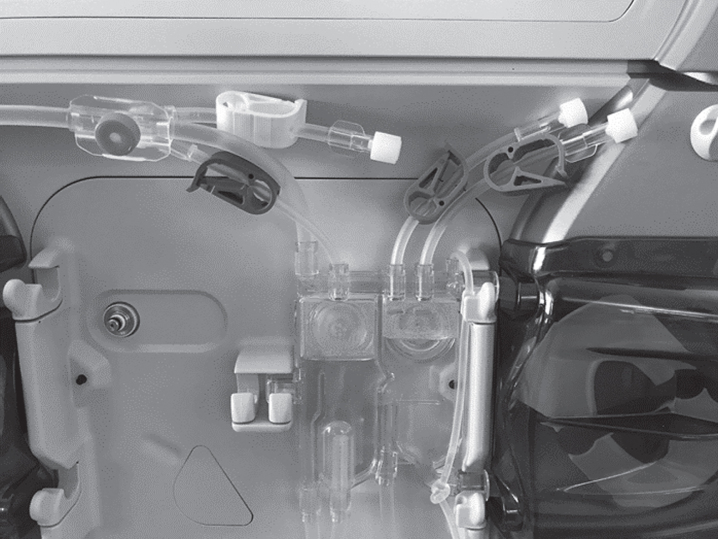
Examples of typical clamps on the bloodlines.

The nurses perceived that the size and type of material used for the clamps on the bloodlines made a difference in how stiff they were, and thus required more or less force when opening and closing them. When the clamps were larger, the nurses complained of an increased musculoskeletal load and strain on their thumbs and fingers. Examples of the clamps are shown in Fig. 4. Sometimes a two-handed grip was required to close the clamps. After starting the haemodialysis treatment, all of the bloodlines and dialysate hose connections were checked several times. Properties in the bloodlines change with the change in temperature from the warm blood. Because of this, the nurses needed to retighten a majority of the connections multiple times during the dialysis to ensure they would not leak. The nurses experienced a strain on their fingers and wrists when doing that.

The electronic monitoring of the dialysis treatment could be programmed so that the nurse must log in to change the settings on the machine. To log in, a nurse would have to raise their arm to enter their password on the touch-screen monitor. The number of taps on the screen depended on how long the password was. During one nurse’s workday (07:00–15:00), 453 taps on the screen were counted. The nurses experienced this as tiring for their shoulders and neck. Having to hold their arms up over shoulder height to work on the screen often led to perceived numbness or pain in the trapezius muscles and arms.

After the dialysis treatment was concluded for patients with an AVF or AVG, compression was needed to form a clot and stop the bleeding at the cannulation sites. This was usually performed by the nurse, and required steady pressure applied by 2–4 fingers. The applied pressure was not hard, but the static pressure on the AVF/AVG was still stressful for the body since it took about 5–20 min. The nurses experienced a strain was put on their fingers, arms and shoulders when applying pressure on the cannulation sites. They also perceived a strain was put on their backs due to the twisted position they had to assume while sitting or standing. The need to have physical access to the cannulation sites reduced the possibility for an optimal ergonomic body position.

Cleaning after a completed dialysis treatment involved working in cramped spaces between the dialysis machine and e.g. the bed, treatment chair, walls and water pipes. The nurses’ work positions varied from having to stand on their toes to having to squat in order to reach everything. The dialysis machines have protruding parts e.g. knobs and holders, which could cause damage to the nurses’ hands and skin on their wrists.

The gathering of materials and supplies could give pain in different parts of their hands depending on the form of the packaging and how it could be lifted out of the shipping carton. A three-finger grip was needed to unpack filters no matter if they were upright or laying down. This work also included moving large and small containers of concentrated dialysate unless a central delivery system existed. Consumable materials needed to be moved and distributed from the storage room to the dialysis rooms. Even the sorting of packaging materials for recycling was describe as physically demanding.

The physical space surrounding the patient during the dialysis treatment could be quite limited. There were often many dialysis machines in the same room. The design of the premises and the location of the equipment in relation to the patient’s blood access could require the repeated movement of the dialysis machines during the day. Shifting the machinery or other equipment depended on whether the patient was in a bed or treatment chair and on which side the patient had their access. However, the observations revealed that the routines for moving equipment varied substantially in the different dialysis centres. Machines at one centre were moved as needed for each patient, while machines at other centres were only moved if they needed service. The nurses said that the machines were physically heavy to move. The connections that are required for the machine to function e.g. power cords and water hoses made movement more difficult. These movements were often done in unfavourable positions that resulted in a noticeable strain on the torso and back muscles, and in some cases, even the arms.

## 4 Discussion

The explorative study we conducted provided an insight into the work situation of haemodialysis nurses from an ergonomic perspective. The study revealed eight work activities that could increase the risk of developing work-related musculoskeletal problems. A particular work task that the nurses associated with thumb, index finger and middle finger complaints was the repetitive flushing of the vascular accesses. According to the KDOQI clinical practice guideline for vascular access [13], the routine flushing of the lumens with normal saline using a turbulent flushing technique is considered standard practice to maintain vascular access patency. The guideline does not give any recommendation on the number of flushes needed to clear the vascular access of blood or fibrin build-up. The haemodialysis nurse is instead expected to use their best clinical judgment in deciding the quantity of the recurrent flushes. In our observations, a 10 ml syringe was typically used for flushing with up to 15 flushes per lumen. The size of the syringe and the frequency of the flushing every workday could generate a significant cumulative strain on the hand, and therefore further exploration is warranted. Previous studies have concluded that repeated nursing tasks [8] with repeated movement of the wrist or fingers for more than four hours during a workday are associated with the development of wrist or hand pain [4, 9]. As the risks for disabling wrist and hand pain seem to be induced by as of yet unidentified factors [4], the flushing procedures impact on the development of musculoskeletal problems needs to be investigated in future studies.

The finding that nurses experienced fatigue in their fingers and the musculature and tendons on the back of their hand after priming several machines, confirms the importance hand complaints should have as an area of focus for both nurses and managers. It suggests that an emphasis should be placed on preventing priming related hand complaints when designing and planning the daily workflow. Previous research has revealed that there is a significant difference in the required number of twisting/turning movements performed during the priming procedures with the different machine types, but this does not seem to have a relationship to the development of hand complaints among haemodialysis nurses [10]. An attention-grabbing finding derived from the reflective dialogue was that the exact site(s) of the hand complaint(s) varied depending on which dialysis machine was used. This suggests that the design of the machine and the subsequent priming procedures cause different physical loads and therefore affect different anatomical structures in the hand. What that previous research did not account for were these differences. The measurements used could not detect in enough detail regarding which part(s) of the hand had been affected. Strategies for preventing musculoskeletal complaints among nurses includes adequate staffing, education, manual handling policies, manual handling training and the development of a culture of occupational safety [14]. Despite the well-documented prevalence of musculoskeletal complaints among nurses [1, 2], evidence supporting interventions that can reduce work-related injuries or pain is still lacking [15].

The experienced resistance when opening and closing the clamps on the bloodlines was described by the haemodialysis nurses’ as giving discomfort or pain in the thumbs and fingers. Although the nurses expressed potential differences between various manufacturers, in the study by Westergren et al. [10], no significant differences in the number of clamping grips used were found. Similarly, the act of connecting the patient’s access to the dialysis machine’s bloodlines also generates a certain resistance with the rotational movements. After performing these tasks several times every workday, haemodialysis nurses experienced fatigue in various parts of their hands. The frequency of certain nursing tasks is known to increase the likelihood of negative effects on wrists and hands [8]. In addition to these fine motor tasks, haemodialysis nurses are also required to perform manual handling. This may involve the movement of patients, heavy and clumsy dialysis machines that can weigh100–130 kg, beds or treatment chairs, and also the removal of material used for the treatment. Since the workspace around the equipment can be limited, it is reasonable to assume that the manual handling involves a greater ergonomic risk for the development of musculoskeletal complaints. Limited workspaces are known to prevent the application of injury prevention techniques [14].

Nurses in general typically perform their patient related work tasks in a standing position [16]. This also applies to nurses in the haemodialysis setting. All of the tasks involved in the setting up/priming of the dialysis machines are done in a standing position. During the dialysis treatment, the tasks are also performed in the standing position with the exception of the tasks related to the handling of AVFs or AVGs. The nurses can either sit or stand depending on patient related prerequisites and/or personal preference. From an ergonomic perspective, it is important to acknowledge the mandatory manual compression on the AVF or AVG at the end of each haemodialysis session [13]. This task is virtually always associated with a static working position accompanied by an awkward body posture that can last up to 20 min per patient. As it is common for the nurse to be responsible for more than one patient each shift, this task has the potential to have an important impact in the ergonomics of the workday. How this affects the development of musculoskeletal complaints is unknown and therefore warrants further investigation.

According to Warnakulasuriya et al. [17], is it unusual for nurses to work with their hands above shoulder height. Our findings suggest that this is not always the case for nurses working in the haemodialysis setting. Since the frequent tapping on the touch-screen monitor situated upper most on the dialysis machines (the observed heights of the machines were about 1.5–1.6 m) was cited as a something that induced discomfort in the nurses’ hands and trapezius muscles, it could be an unforeseen occupational hazard. Due to the placement of the screens, nurses of a short stature can experience greater musculoskeletal loads than taller nurses. Since the position of the screen cannot be adjusted higher or lower, there will be inherent inequalities in the ergonomic working conditions of haemodialysis nurses.

Many different work tasks are performed by nurses during the daily operation of the haemodialysis centres [18, 19]. Only some of these tasks are described and discussed here since the focus of the study was to explore potential occupational hazards that could lead to musculoskeletal complaints. This limits the transferability of our findings. Moreover, the risk exposure level of each work task has not been rated, which will be needed in future studies. While there was diversity in key demographic variables, other characteristics of our sample place some limits on the generalizability of the findings. The nurses who were included were self-selected from the employees who worked on the day of data collection. Since the data were collected on two or three occasions at each centre, all of the nurses theoretically had the possibility to participate. When conducting observations and making field notes, it is important to acknowledge the influence of subjectivity in the research process [12]. Since the observer in our study did not have any personal experience working in a haemodialysis setting, the risk for subjectivity is negligible. Additionally, the unintentional communication of any expectations would be similarly negligible.

## 5 Conclusion

Our findings add to the understanding of the work-related factors that contribute to musculoskeletal complaints among haemodialysis nurses. The findings support the hypothesis that there might be an association between materials used for haemodialysis and the development of work-related musculoskeletal hand complaints in this population. Repetitive work tasks that have an impact on the development of musculoskeletal problems need to be further investigated.

## Funding

This study was financially supported by research grants from AFA Insurance (AFA reg. no. 170075) and the University of Gävle, Sweden.

## Conflict of interest

No conflict of interest has been declared by the authors.
